# Retraction: Nanocurcumin: preparation, characterization and cytotoxic effects towards human laryngeal cancer cells

**DOI:** 10.1039/d6ra90021f

**Published:** 2026-02-11

**Authors:** Demiana H. Hanna, Gamal R. Saad

**Affiliations:** a Department of Chemistry, Faculty of Science, Cairo University Giza 12613 Egypt dr_demiana@yahoo.com

## Abstract

Retraction of ‘Nanocurcumin: preparation, characterization and cytotoxic effects towards human laryngeal cancer cells’ by Demiana H. Hanna and Gamal R. Saad, *RSC Adv.*, 2020, **10**, 20724–20737, https://doi.org/10.1039/D0RA03719B.

The Royal Society of Chemistry hereby wholly retracts this *RSC Advances* article due to concerns with the reliability of the data.

Concerns were raised with the reliability of the flow cytometry analysis in Fig. 7B. The authors were contacted and asked for a response to the concerns and the raw data. The authors provided processed results that they said were generated at the time of acquisition and derived directly from the original instrument readings, but they were unable to provide the original Flow Cytometry Standard files.

An independent expert has confirmed that the published data shows signs of manipulation, and the data provided by the authors does not address the concerns raised.

Given the significance of these concerns, the Editor has lost confidence that the findings presented in this paper are reliable.

The authors were informed about the retraction of the article. Demiana H. Hanna and Gamal R. Saad do not agree with the decision.

Signed: Laura Fisher, Executive Editor, *RSC Advances*

Date: 4th February 2026

